# METABOLIC INTEGRITY – A FACTOR IN AGING AND A PLAYER IN THE MECHANISMS OF CR

**DOI:** 10.1093/geroni/igz038.262

**Published:** 2019-11-08

**Authors:** Rozalyn Anderson

**Affiliations:** University of Wisconsin Madison, Madison, United States

## Abstract

An emerging paradigm in aging research identifies metabolic dysfunction as a root cause in age-related disease vulnerability. Several diseases of aging, including diabetes, cancer, and neurodegeneration, have an established metabolic component. Our studies have focused on links between metabolic status and disease vulnerability. Caloric restriction (CR) delays aging and the onset of age-related disease in diverse species, including nonhuman primates. Molecular profiling identifies CR responsive elements in the transcriptome, proteome, and metabolome that are highly enriched for metabolic pathways and in particular mitochondrial processes. These data show that improvements in health and survival are associated with maintenance of system wide metabolic homeostasis and preserved energy metabolism among tissues. Metabolic biomarkers identified in these studies may be clinically relevant for the early identification of elevated disease risk in humans and could even be potential targets for the development of novel strategies to lower disease vulnerability as a function of age.

